# High electromechanical strain and enhanced temperature characteristics in lead-free (Na,Bi)TiO_3_–BaTiO_3_ thin films on Si substrates

**DOI:** 10.1038/s41598-018-26309-4

**Published:** 2018-05-18

**Authors:** Yoshiaki Tanaka, Shoji Okamoto, Kazuya Hashimoto, Ryoichi Takayama, Takakiyo Harigai, Hideaki Adachi, Eiji Fujii

**Affiliations:** 1 Engineering Division, Automotive & Industrial Systems Company, Panasonic Corporation, 1006 Kadoma, Kadoma City, Osaka, 571-8501 Japan; 2 Advanced Research Division, Panasonic Corporation, 1006 Kadoma, Kadoma City, Osaka, 571-8501 Japan

## Abstract

Here, we demonstrate the high electromechanical strain and enhanced temperature characteristics in the *c*-axis-oriented lead-free (Na,Bi)TiO_3_–BaTiO_3_ (NBT–BT) polycrystalline thin film prepared on Si substrates by rf magnetron sputtering. The effective transverse piezoelectric coefficient, *e*_31_^*^, estimated from the electromechanical strain measured under high electric field, reaches a high level of −12.5 C/m^2^, and is comparable to those of conventional Pb(Zr,Ti)O_3_ films. *In-situ* X-ray diffraction measurement and electron diffraction analysis revealed the electromechanical strain of the NBT–BT film to originate predominantly in elongation of the tetragonal (*P4bm*) crystal lattice in the *c*-axis direction. In addition to the large *e*_31_^*^, the NBT–BT film exhibits enhanced permittivity maximum temperature, *T*_m_, of ~400 °C and no depolarization below *T*_m_, as compared to bulk NBT–BT having *T*_m_ ≈ 300 °C and a depolarization temperature of ~100 °C. We conclude that the enhancement of temperature characteristics is associated with the distorted *P4bm* crystal lattice formed by deposition-induced stress and defects. We believe that the present study paves the way for practical applications of lead-free piezoelectric thin films in electromechanical devices.

## Introduction

Piezoelectric thin films are integral parts for actuation and sensing in micro-electromechanical systems (MEMS)^1^. Much attention has been paid to lead-based material, Pb(Zr,Ti)O_3_ (PZT), for such applications due to its large piezoelectric properties and stable temperature properties. At the present day, the major efforts for realizing high-performance thin films of PZT on Si wafers have enabled the practical use of piezoelectric-MEMS devices like actuators for inkjet head printers and as angular rate sensors^2^. The next challenging step in the research field of piezoelectric materials, including thin films, is likely to be the development of lead-free alternatives with high piezoelectricity and temperature stability comparable to that of PZT films to be able to meet increasing worldwide environmental concerns about harmful lead in PZT films. Our basic approach to achieving this goal is to utilize the particular properties of thin films that sometimes appear in the course of the film deposition process. Typical examples include internal stress and internal bias fields, both of which can stabilize ferroelectric ordering, thereby enhancing both piezoelectricity^3,4^ and temperature stability^5–8^.

Complex solid-solution (1 − *x*)(Na_0.5_Bi_0.5_)TiO_3_–*x*BaTiO_3_ (NBT–BT) comprising rhombohedral (Na_0.5_Bi_0.5_)TiO_3_ and tetragonal BaTiO_3_ has been identified as one of the most promising candidates for lead-free piezoelectric materials, since it shows a large piezoelectric coefficient at compositions near the morphotropic phase boundary (MPB) of *x* = 0.06–0.07^9,10^. The ceramic NBT–BT is reported to show the longitudinal piezoelectric coefficient, *d*_33_, reaching 100–200 pC/N^9,11^, which reportedly represents a twofold or more improvement over [001]-textured ceramics^12,13^. For the single crystal NBT–BT poled along the [001] direction, *d*_33_ and transverse piezoelectric coefficient, *d*_31_, attain 483 pC/N and −115 pC/N^14^, respectively. Unfortunately, the piezoelectric properties of NBT–BT with a composition close to the MPB disappears suddenly at the depolarization temperature, *T*_d_, of close to 100 °C^9,11^, well below the permittivity maximum temperature, *T*_m_. The presence of *T*_d_ restricts the potential use of NBT–BT at elevated temperature. Structural investigations for NBT–BT have revealed that the unpoled bulk NBT–BT has different phase relations from those of the poled one, consisting of a weakly polar ferrielectric tetragonal phase with a very slight tetragonality, with a *P4bm* space group over *x* = 0.06–0.10 compositions, whereas the poled one is comprised of the strongly polar ferroelectric phase^15,16^. The phase with a *P4bm* space group is recently reported to reversibly/irreversibly transforms into the strongly polar ferroelectric tetragonal phase with *P4mm* space group on applying an <100> directional electric (*E*) -field more than a threshold^16–19^. This phase transition is accompanied by a large lattice elongation/polarization extension, resulting in a very high electromechanical strain (*d*_33_^*^ reaches up to 2500 pm/V)^18^.

Recently, we demonstrated that sputtered NBT–BT films on MgO substrates exhibit much larger piezoelectric coefficients than those of PZT films: the highest *d*_31_^*^ reaches −221 pm/V^20–23^. The most important task for integrating the NBT–BT to MEMS devices is to deposit the high-performance films onto Si substrates that can be applicable to MEMS processes^1^. However, the NBT–BT films on Si are generally in tensile stress arising from lower thermal expansion coefficient, *α*, of NBT–BT (*α* = 11 ppm/K)^21^ comparing that (*α* = 2.6–4.3 ppm/K) of Si, which causes a distortion of the crystal lattice toward the in-plane (*a*-axis) direction i.e. *a*-axis preferential orientation and thereby the piezoelectric properties become low ^23–25^.

In this study, we successfully prepare the NBT–BT polycrystalline thin film with a *c*-axis preferential orientation on (100)-LaNiO_3_ (LNO)-buffered Si substrates by rf magnetron sputtering method. The *c*-axis orientation is realized by application of intrinsic compressive stress through energetic particle bombardment during sputtering. The obtained film shows a high electromechanical strain with *e*_31_^*^ = −12.5 C/m^2^ and enhanced temperature characteristics wherein the *T*_m_ is increased to ~400 °C from ~300 °C seen in bulk NBT–BT and no depolarization occurs below *T*_m_. We also discuss the origin of the properties of NBT–BT film, especially based on the film’s individual properties as related to the deposition process.

## Results and Discussion

### *c*-axis oriented polycrystalline thin film of NBT–BT on Si

**Figure 1:**
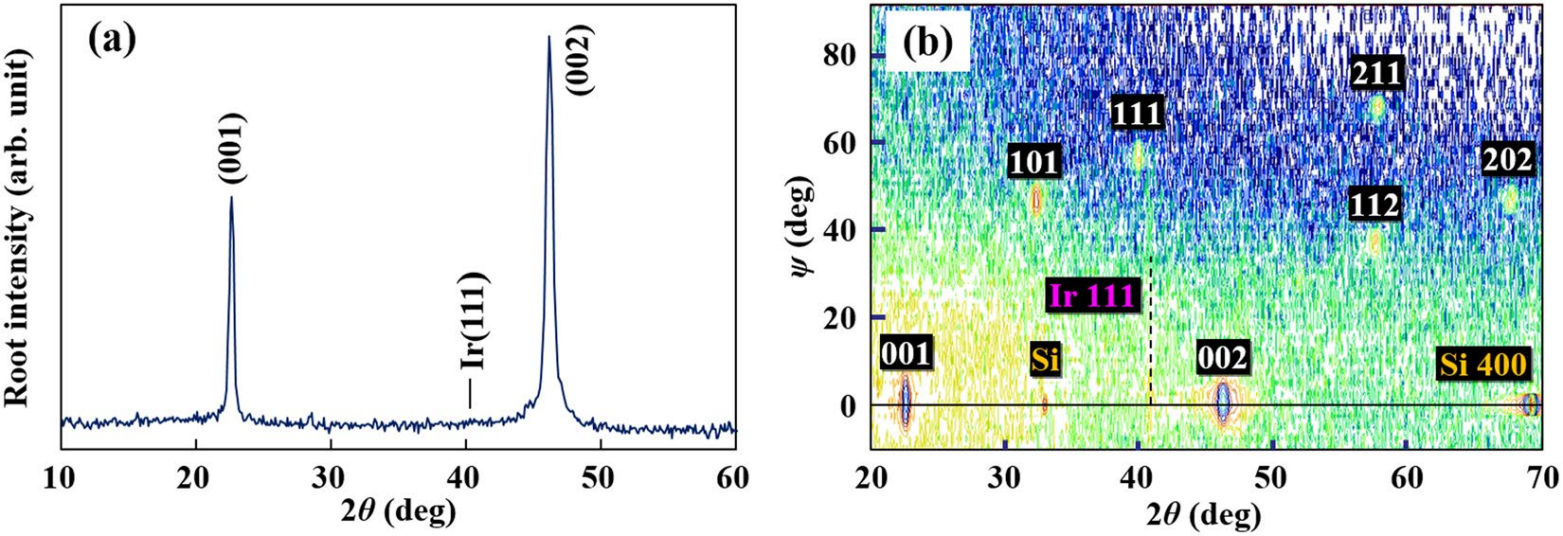
(**a**) *θ*–2*θ* XRD pattern and (**b**) *ψ*–2*θ* XRD map for the NBT–BT thin film. The dashed line in (**b**) denotes a diffraction peak assigned to Ir.

**Figure 2:**
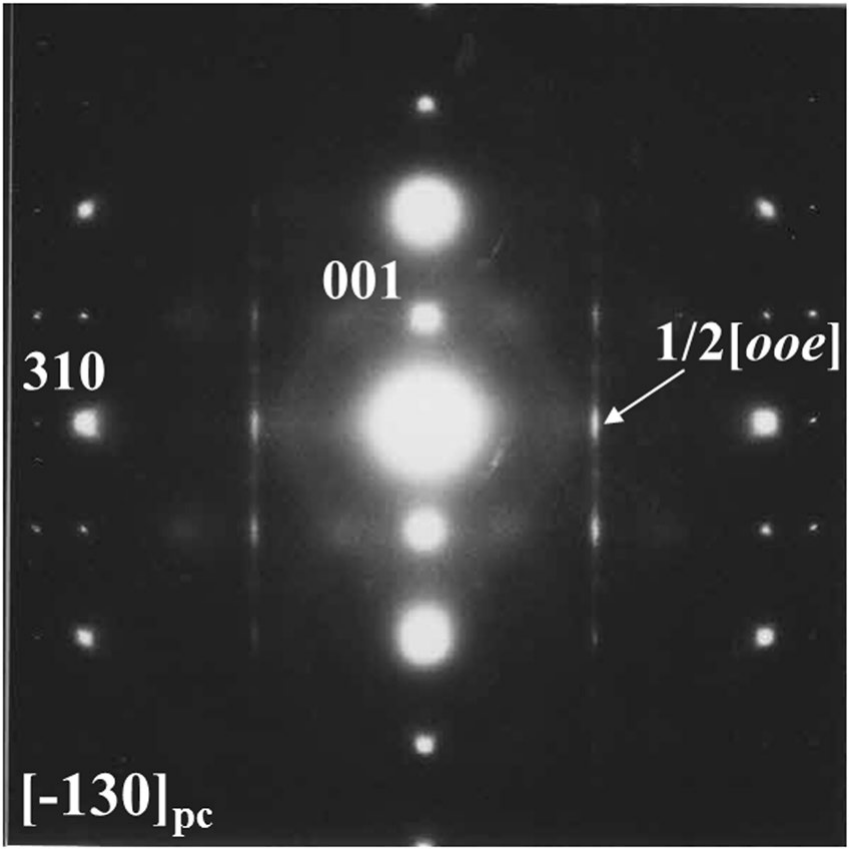
ED image for the NBT–BT thin film. The incident beam is along the [−130]_pc_ direction. The arrow indicates the 1/2[*ooe*] super-lattice diffraction spot.

Figure 1(a) shows the out-of-plane X-ray diffraction (XRD) pattern for the (1 − *x*)NBT–*x*BT (*x* = 0.07) film on Si (100) with the intermediate LNO/Ir electrode. It is confirmed that the NBT–BT film has a single perovskite phase with a preferential orientation in the <100> direction normal to the substrate. The XRD *ψ*–2*θ* map (Fig. 1(b)) for the same sample revealed that the crystal orientations of the NBT-BT grains were rotated randomly in the in-plane direction and traces of secondary phases were hardly observed. Unlike for PZT films^26^, no splitting of the out-of-plane XRD peaks was seen, indicating that coexistence of *a*- and *c*-domains or differently-structured phases did not take place in the NBT–BT film. The pseudocubic (pc) in-plane and out-of-plane lattice parameters, estimated from the (101) and (001) XRD peaks, were *a*_pc_ = 0.391 nm and *c*_pc_ = 0.393 nm, respectively, for the NBT–BT film. These structural results demonstrate that the NBT–BT film is of *c*-axis preferred orientation with a single tetragonal lattice that shows tetragonal distortion (*c*/*a*)_pc_ value of 1.004. The *c*/*a* value of the present film is much smaller than that (~1.014) of the poled bulk NBT–BT samples with the same BT content^27^. Morphological texture of the NBT–BT film was found to have a smooth surface and a dense structure with columnar grains by using atomic force and electron microscopy (see Supplementary Fig. S1a,b). For the detailed investigation on crystal structure of the NBT–BT film, electron diffraction (ED) measurement was carried out using a transmission electron microscope (TEM). Figure 2 shows the ED image obtained along the [−130] incident beam direction of the film. The diffused 1/2[*ooe*] super-lattice diffraction spots (*o* and *e* denote odd and even Miller indices, respectively) with streaking parallel to the [001] direction was observed in the whole grains of the film. This feature is a characteristic of bulk NBT–BT samples with a *P4bm* space group (hereinafter referred to as the *P4bm* phase)^28,29^, being the 1/2[*ooe*] super spot attributed to its in-phase oxygen octahedron tilts in perovskite cell^30^. This ED result evidences that the NBT–BT film mainly consists of the *P4bm* phase.

### Ferroelectric polarization – *E*-field (*P*–*E*) hysteresis and high electromechanical strain

**Figure 3:**
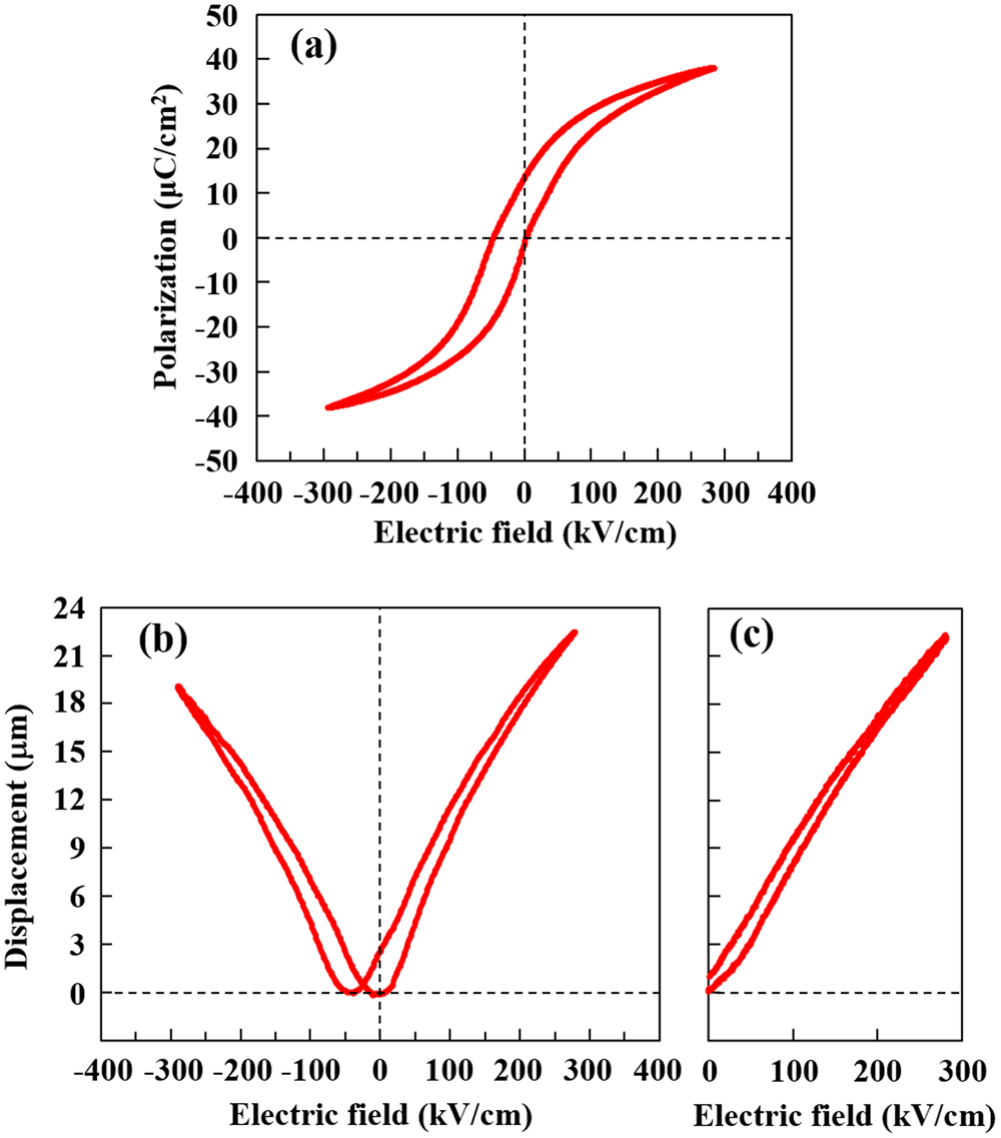
Ferroelectric properties and electromechanical strain. (**a**) *P*–*E* hysteresis loop and *δ*–*E* curves obtained by (**b**) bipolar and (**c**) unipolar field cycling for the NBT–BT thin film.

Figure 3(a) shows the *P*–*E* hysteresis loop of the NBT–BT film recorded at 10 Hz in a metal–insulator–metal electrode structure with an Au (100 nm) top and LNO (200 nm) bottom electrodes. The NBT–BT film exhibited a quite slim ferroelectric hysteresis loop with a remnant polarization, *P*_r_, of 13.3 μC/cm^2^. Also seen is that the hysteresis loop shifted along the negative field axis, revealing an internal bias field across the film. The observed internal bias field of about –25 kV/cm is comparatively higher than those observed in some previously reported NBT-based films^31,32^. An internal bias field is occasionally observed in ferroelectric films when they are deposited under high-bombardment conditions^33^ or low-oxygen-partial-pressure conditions^34^, or if they have a compositional gradient^35^ as well as asymmetric electrodes^36^: it is thought to be attributable to the accumulation of charged defects such as oxygen vacancies into interfaces^37^, alignment of vacancy-related defect dipole complexes^38^, strain gradients across the film^39^ and so on. We considered that the internal bias field in the present NBT–BT film is most likely to result from deposition-induced defects, including defect dipoles, judging from the fact that the present film is deposited under high bombardment conditions during sputtering for the *c*-axis orientation onto a Si substrate. Herein, the asymmetric electrode structure of the NBT–BT film, i.e., Au/NBT–BT/LNO, was confirmed not to be the cause of the voltage shift, because the same voltage shift was yielded with a symmetric electrode structure, i.e., LNO/NBT–BT/LNO.

To investigate the piezoelectric properties of the NBT–BT film, displacement–*E*-field (*δ*–E) curves were measured using both bipolar and unipolar *E*-field cycling for the unimorph cantilever beams. The bipolar *δ*–*E* curve for the NBT–BT film, given in Fig. 3(b), showed a butterfly-like shape along with a negative voltage shift corresponding to the *P*–*E* hysteresis loop. The effective transverse piezoelectric coefficient, *e*_31_^*^, which was calculated with the linear component of the *δ*–*E* curve measured at high *E*-field as described elsewhere^20^, was as large as −12.5 C/m^2^ for the NBT–BT film. The *e*_31_^*^ value of the NBT–BT film is comparable to those of conventional PZT films^1,2^. Herein, the effective piezoelectric strain coefficient, *d*_31_^*^, calculated in the same manner as in the previous works^21^ was –135 pm/V. It should be noted that if the *a*-axis orientation is preferred, i.e. *c*/*a* is less than unity, for the NBT–BT film, we confirmed the *e*_31_^*^ to get worse together with decreasing *P*_r_, this tendency being in accordance with the results of our previous study for NBT–BT films on various substrates with different *α*^22,23^. The fact described here represents the impact of the *c*-axis orientation in the NBT–BT films. Regardless of the high electromechanical strain in the NBT–BT film, the dielectric permittivity, *ε*_r_, which is estimated to be 726, is not much higher than conventional piezoelectric materials. This is apparently due to the presence of the internal bias field and/or the lower contribution from domain-wall-motion, which primarily determines the *ε*_r_ of ferroelectrics^40^. It should be also pointed out here that the electromechanical strain yielded in the unipolar excitation of the NBT–BT film exhibited a small hysteresis and a relatively high linearity [see, Fig. 3(c)], both of which, along with the large amount of strain, are an important property for actuator applications. We note, however, that in the low *E*-field region, the linearity of the electromechanical strain for the NBT–BT film decreases as discerned in the unipolar strain during *E*-field loading [Fig. 3(c)], which means that the *e*_31_^*^ of the NBT–BT film drops at low *E*-field. Therefore, applications of the NBT–BT films are basically limited to large strain actuators operated at high *E*-field such as an inkjet printer head, whereas applying dc electric bias to the film leads to improve the low *E*-field nonlinearity. This kind of behavior of the NBT–BT film is different from the PZT films showing high linear strain even at low *E*-field. Instead, the characteristics of the present NBT–BT film are rather similar to those of the lead-based relaxor films such as Pb(Mg,Nb)O_3_−PbTiO_3_ (PMN−PT) films, since both the films exhibit a slim *P*–*E* hysteresis with rapid reduction of the polarization during *E*-field unloading as well as a large electromechanical strain with nonlinearity at low *E*-field^4,41,42^. Detailed comparison of the two films will be described in the last section of this paper.

### *In-situ E*-field dependent XRD experiments

**Figure 4:**
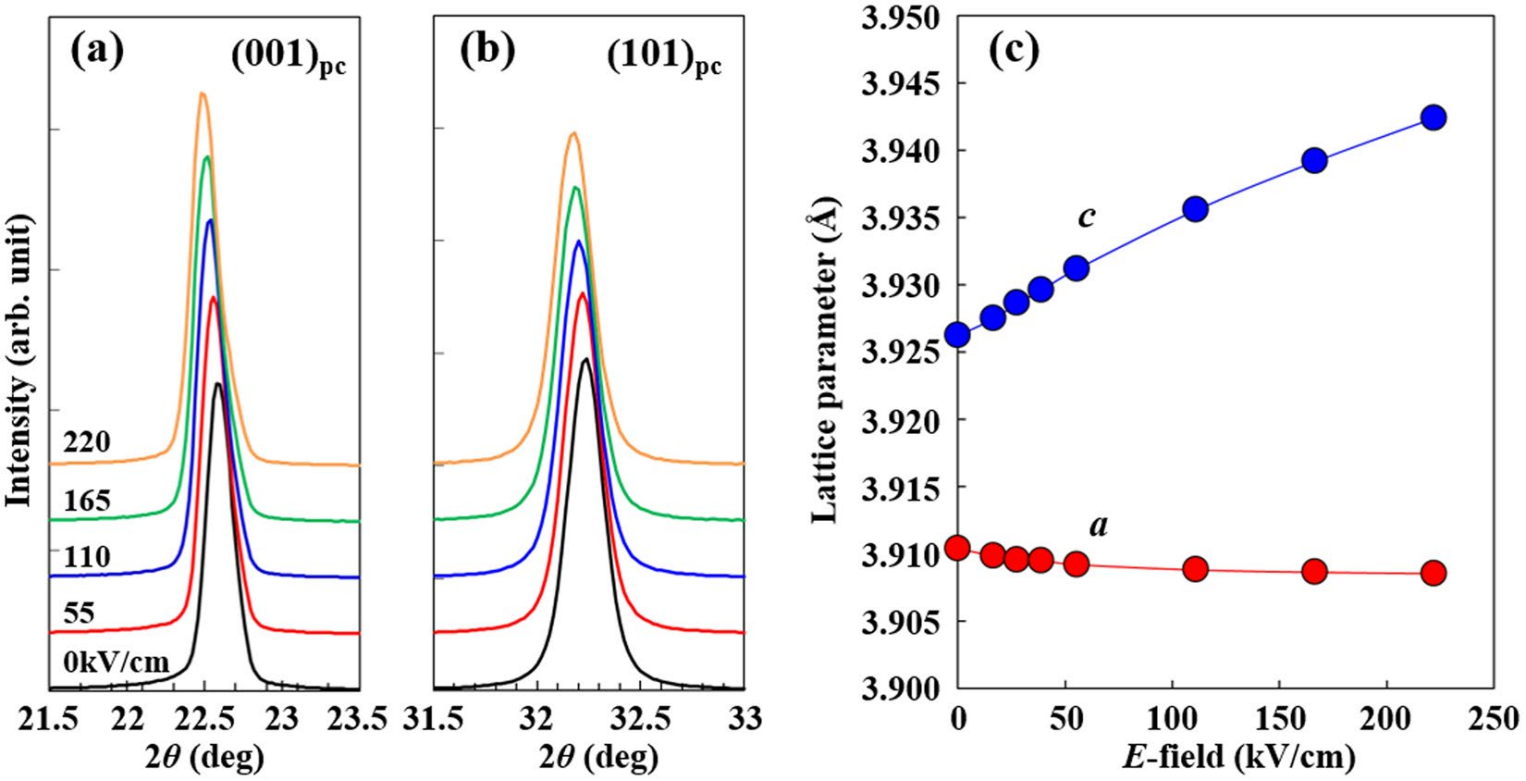
*In-situ E*-field-dependent XRD results for the NBT–BT thin film. (**a**) XRD peak of (001)_pc_ and (**b**) (101)_pc_ planes under various *E*-field//[001]_pc_. (**c**) *E*-field-dependent lattice parameters *a* and *c* calculated from (001)_pc_ and (101)_pc_ peak positions.

Our XRD analysis under no external *E*-field revealed the NBT–BT film to possess a single tetragonal crystal lattice with a small tetragonality. In this study, we examined the *E*-field-dependent crystal lattice by means of *in-situ* XRD experiments to probe the contribution of the crystal lattice to electromechanical strain. Figure 4(a,b) respectively show *E*-field dependent (001) and (101) XRD peaks for the NBT–BT film. The applied *E*-field increased a continuous peak shift toward lower angle direction: this was observed for both the peaks, indicating an increase in *d*-spacing. Also observed is that splitting of the peaks corresponding to the *a*/*c* domain formation did not take place on applying the *E*-field. Figure 4(c) shows the lattice parameters *a* and *c*, estimated from the (001) and (101) peaks, plotted against the applied *E*-field. The *c* parameter increased in a linear manner with increasing *E*-field, whereas the *a* parameter decreased straightforwardly, but also showed a trend toward saturation at higher *E*-fields. This saturation can be understood as a result of the film being clamped to a rigid substrate. From the slope of the *c* parameter versus the *E*-field plot, we calculated an estimate for the effective longitudinal piezoelectric coefficient, *d*_33_^*^_,_ with *d*_33_^*^ = *∆c*/*c* ∙1/*E*, where *∆c* is the difference in *c* parameters before and after applying the *E*-field, resulting in a large *d*_33_^*^ value reaching about 200 pm/V. It should be emphasized that the NBT–BT film is laterally perfectly clamped. In this approximation, the *d*_33_ of film, *d*_33_,_*f*_, is given by^43^:

1 $${d}_{33,f}={d}_{33}-\frac{2{s}_{13}^{E}}{{s}_{11}^{E}+{s}_{12}^{E}}{d}_{31}$$

where *s*^*E*^ is the compliance at constant field with the subscripts denoting the stress and strain directions. This equation means that *d*_33_ of a film is always smaller than that of the unclamped bulk; the *d*_33_,_*f*_ is nearly one half of the *d*_33_^44^, so that the unclamped *d*_33_^*^ of the NBT–BT film ought to rise to a high ~400 pm/V. Taking into account the fact that *d*_33_ is generally larger, by a factor of approximately 2 or 3, than *d*_31_ for piezoelectric or electrostrictive materials, respectively^45^, the NBT–BT film’s *d*_33_^*^, evaluated from the *in-situ* XRD, is large enough to explain the transverse value (*d*_31_^*^ = −135 pm/V) from the macroscopic *E*-field-induced strain in the cantilever beam (*δ*-*E* curve). These XRD analysis results demonstrate that the macroscopic electromechanical strain of the NBT–BT film is dominated by the elongation of the crystal lattice in the *c*-axis direction, rather than by non-180°-domain wall motion, which is commonly thought to be the main cause of the high electromechanical strain seen in piezoelectric materials. This conclusion supports the film’s abovementioned relatively low permittivity.

### Difference between the film and bulk *P4bm* phases and possible reason for the large lattice elongation of the film

The structural analysis results demonstrated the present NBT–BT film to be comprised of the *P4bm* phase. The *P4bm* phase of bulk NBT–BT possesses ferrielectric nature, which is recognized as a double hysteresis loop in *P*–*E* relation when the *P4bm*-to-*P4mm*-phase transition occurs reversibly^17–19^. On the one hand, the *P4bm* phase of the NBT–BT film seems to be governed by ferroelectric, rather than ferrielectric nature [see, Fig. 3(a)], and the *P*_r_ value (13.3 C/cm^2^) of the film is clearly large as compared to that of bulk *P4bm* phase. The induction of this large *P*_r_ is likely to be linked to the fact that the crystal lattice of the *P4bm* phase of the film is distorted to a greater extent along the *c*-axis direction compared than in the bulk NBT–BT, i.e., the (*c*/*a*)_pc_ of the film is 1.004, whereas the most accepted value for bulk is less than 1.001^46,47^. It is most likely that the distortion of the crystal lattice results not only from compressive stress caused by energetic particle bombardment in sputtering but also from deposition-induced defects such as defect dipoles^48^. The latter is evidenced by the presence of the internal bias field^38^, and it can directly contribute to the increase in *P*_r_. The fact that the NBT–BT film does not show the ferrielectric behavior indicates that the *P4bm*-to-*P4mm*-phase transition like bulk is not applicable to the NBT–BT film. Indeed, the NBT–BT film exhibits linear variations in the lattice parameters with respect to the *E*-field [see, Fig. 4(c)], whereas bulk NBT–BT shows discontinuous lattice parameter change from psudocubic axes into separated tetragonal *a* and *c*-axes at the threshold *E*-field where the phase transition take places^17,49^. The *E*-field dependent lattice parameter variation also indicates that the phase transition does not occur clearly in the NBT–BT film. The absence of obvious phase transition may be attributed to the clamping effect of the underlying substrate. The phase transition of the *P4bm* phase does in fact require *a*-axis contraction to release the oxygen octahedron tilts, and hence it should be markedly affected by in-plane clamping. From these results, we may conclude that the large *E*-field induced lattice elongation in the NBT–BT film is originated from the characteristic feature of the *P4bm* phase itself, rather than its phase transition like the case of bulk NBT–BT. It is interesting to note here that in bulk NBT–BT reported by Ge *et al*.^18^, the *P4bm* phase itself shows high electromechanical strain (*d*_33_^*^ = 350 pm/V) under an *E*-field below the threshold *E*-field. Fortunately, the absence of any obvious phase transition in the NBT–BT film makes it possible to actualize the low-hysteretic unipolar electromechanical strain with a better linearity than for bulk NBT–BT [Fig. 3(c)]. The internal bias field built into the NBT–BT film during the deposition would also contribute to the linearity due to an increase in *P*_r_.

### Enhanced temperature characteristics

**Figure 5:**
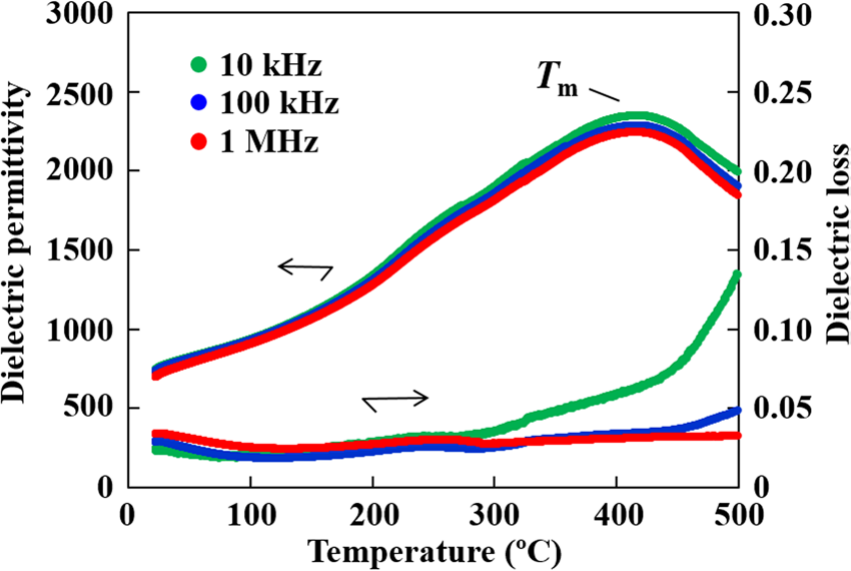
Temperature-dependence of dielectric permittivity and loss for the NBT–BT thin film. The data were taken during heating.

For the evaluation of the temperature characteristics of the NBT–BT film, the dielectric properties were measured with respect to temperature, *T*, with various measuring frequencies (10 kHz−1 MHz). Figure 5 gives the temperature-dependence of dielectric permittivity, *ε*_r_, and dielectric loss, tan *δ* for the NBT–BT film. As seen in the *ε*–*T* curve, *T*_m_ of the NBT–BT film lies at a higher temperature (about 400 °C) than for both the poled and unpoled bulk NBT–BT with ferroelectric and ferrielectric nature, respectively, in which *T*_m_ ≈ 300 °C. As for the poled bulk NBT–BT, the other important characteristic temperature, *T*_d_, i.e., the transition temperature from the ferroelectric phase to the ferrielectric phase^9,15^, exists well below *T*_m_ and is generally agreed to become visible as a sharp jump in the *ε*–*T* curve and a clear peak in the tan *δ*–*T* curve^50^. The latter is frequently adopted to gain the exact value of *T*_d_. The aforesaid features related to *T*_d_ were not exactly distinguished in either the *ε*–*T* or tan *δ*–*T* curve in the present NBT–BT film, whereas broad anomalies are seen in both the curves around 250 °C; these anomalies resemble those of unpoled bulk NBT–BT, which can be understood as the relaxor transition temperature or thermal evolution of ferroelectric polar nanoregions^51^, but are not fully identical to them, since its most characteristic feature, i.e., a strong frequency dependence, is not observed in the NBT–BT film.

**Figure 6:**
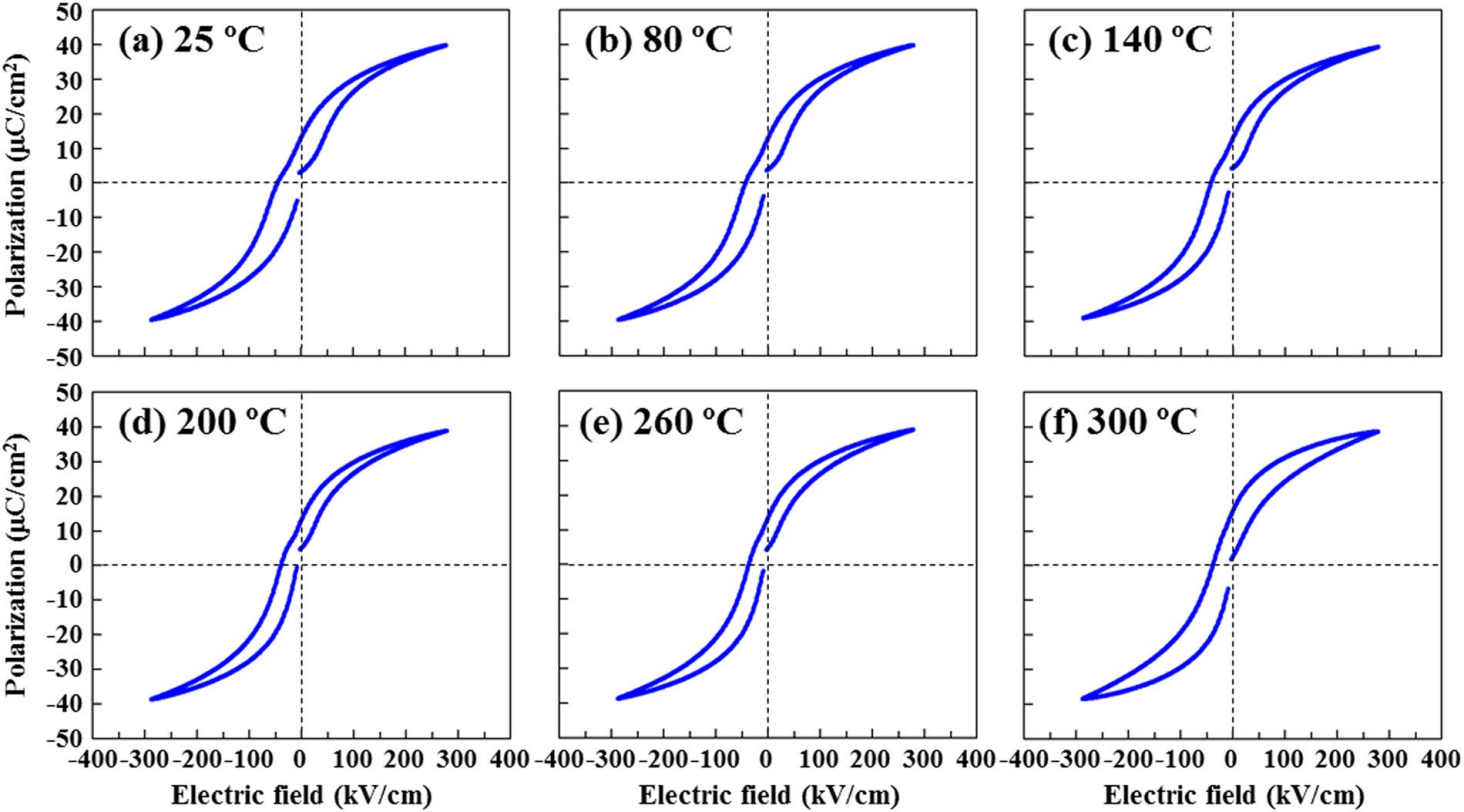
*P*–*E* hysteresis loop measured at different temperatures for the NBT–BT thin film. (**a**) 25 °C, (**b**) 80 °C, (**c**) 140 °C, (**d**) 200 °C, (**e**) 260 °C, (**f**) 300 °C.

To ensure the enhancement of the temperature-dependent dielectric properties of the NBT–BT film, the *P*–*E* hysteresis loop was plotted at 1 kHz with increasing temperatures up to 300 °C, including temperature range where the aforementioned broad anomalies appear. The results showed the polarization value to remain almost unchanged over the measured temperature range (Fig. 6), indicating that the polarization state of the NBT–BT film is fairly stable, whereas the hysteresis loop takes on a bell-like shape at 300 °C due to increased leakage. This fact would mean that the broad anomalies in dielectric properties around 250 °C are irrelevant to the depolarization of the film. The enhanced temperature characteristics of the NBT–BT film, i.e., *T*_m_ = 400 °C and absence of *T*_d_, allow us to predict that the high electromechanical strain of the film will also be maintained at high temperatures. This implies that the temperature range over which NBT–BT film can be used is wider than ferroelectric bulk NBT–BT with *T*_d_ ≈ 100 °C.

### Possible reason for the enhanced temperature characteristics

The findings of this study are that the present NBT–BT film possesses no *T*_d_ and ~30% higher *T*_m_ comparing to the bulk. The absence of *T*_d_ in the NBT–BT film may be related to the fact that the NBT–BT film already has *P4bm* symmetry at room temperature, namely *T*_d_ corresponds to the *P4mm*-phase-to-*P4bm*-phase transition temperature, though showing a ferroelectric behavior rather than a ferrielectric one due to the above-described crystal lattice distortion through the deposition-induced stress. Both the stress and defects give rise to stabilization of polarization of the NBT–BT film, thereby also causing the enhancement of *T*_m_. It should be kept in mind that the *T*_m_ of ferroelectric films is widely agreed to be enhanced by the distortion of the crystal lattice imposed by external stress: for instance, through an in-plane lattice mismatch between the film and the substrate^5,6^. Damodaran *et al*. recently reported the enhancement of *T*_m_ in conjunction with the elongation of the *c*-axis in the internally-biased BaTiO_3_ films prepared by means of pulsed laser deposition, and demonstrated that these features originate from the deposition-induced defect dipoles being ordered along the *c*-axis direction by compressive stress via lattice mismatch^52^. These scenarios appear to be highly applicable to the present NBT–BT film.

### Comparison of NBT–BT film with lead-based relaxor films

From the similarity of the NBT–BT films and the PMN−PT relaxor films, we compared here the characteristics of these two films to demonstrate the originality of the NBT–BT films. The PMN−PT is known as a prototypical relaxor ferroelectrics predominantly exhibiting electrostrictive nature. The NBT-based materials including NBT–BT likewise show electrostriction and relaxor behavior especially below the *E*-field where the phase transition occurs^19,51,53^ but differently from the PMN−PT, having anti-ferro/ferri-ferro-electric ordered nanodomains derived from the *P4bm* phase^15^. On the other hand, as for the present NBT–BT film, the sputtering-derived stress and internal bias field give rise to enhanced polarization leading to the ferroelectric ordering, so that the contribution from electrostriction would become less. This can be evident from the fact that the NBT–BT films have a much better linearity in strain−*E*-field curve compared to that of bulk: the film shows linear relation during *E*-field unloading (see, Fig. 3), whereas the bulk exhibits quadratical relation during both the *E*-field loading and unloading^19,53^. The variation of the linearity can also be recognized in the PMN−PT with different PT content^42^. The most distinctive characteristics of the NBT–BT films compared with the PMN−PT films are the dielectric response depending on temperature: the NBT–BT films have much higher *T*_m_ of ~400 °C and much less frequency dispersion (see, Fig. 5), as compared with the PMN−PT films having *T*_m_ lower than 100 °C and large frequency dispersion^42^. This difference may imply that the NBT–BT films processes more stable and/or larger domains than PMN−PT films. Therefore, the electromechanical property of the NBT–BT films is likely dominated by intrinsic effect, i.e. lattice elongation related to phase transition as mentioned above, rather than the nanodomains which are believed to have a predominant role in lead-based relaxors. However, we do not deny the presence of some kind of domain contribution accompanied by the lattice elongation in the NBT–BT films. Further investigation, such as domain structure observation, is needed in future works.

## Conclusions

A *c*-axis-oriented NBT–BT polycrystalline thin film having a tetragonal *P4bm* lattice was successfully deposited on an LNO-buffered Si substrate by sputtering. The obtained NBT–BT film exhibited high electromechanical strain along with ferroelectric nature, from which the estimated *e*_31_^*^ reaches a high value of −12.5 C/m^2^ at high *E*-field comparable to that of conventional PZT films. *In-situ* X-ray diffraction measurement revealed that the electromechanical strain in the NBT–BT film can be attributed to elongation of the *P4bm* crystal lattice along the *c*-axis direction. The phase with *P4bm* lattice is often observed in the unpoled bulk NBT–BT, but that of the film is more strongly distorted (*c*/*a*~1.004) than in the bulk in the *c*-axis direction due to the deposition-induced stress and defects. The distorted *P4bm* lattice can give rise to improvements in the temperature characteristics within the NBT–BT film, i.e., depolarization does not occur at least until ~300 °C, as compared with ~100 °C for ferroelectric bulk NBT–BT having *T*_d_. Due to the enhanced temperature characteristics, the high electromechanical strain of the film is likely to be maintained at much higher temperatures than bulk NBT–BT. The present study demonstrates this NBT–BT film to have major potential for practical application to MEMS devices.

## Methods

### Film preparation

Rf magnetron sputtering was employed for the preparation of NBT–BT polycrystalline film on an LNO-buffered Si substrate. The LNO layer acts to align the grains of the NBT–BT film in the <100> direction and to enhance its crystalline quality. The film thickness was approximately 2 μm. Detailed preparation procedures were described elsewhere^20,21^. In this study, to alleviate the tensile thermal stress loaded to the film from the substrate and to obtain *c*-axis oriented NBT–BT thin film on Si, we precisely controlled the sputtering conditions. Sputtered films are commonly under compression due to energetic particle bombardment^54–56^, the degree of which is controllable by tailoring the sputtering conditions such as gas pressure, supplied power, and substrate bias^54,55,57,58^. Poling process was not carried out for the obtained film. A sputtering target contained NBT and BT in the MPB composition, and also included a small amount of Mn additive (1 at.%) for suppression of leakage current. Before the deposition of NBT–BT film, Ir (250 nm) bottom electrode was grown on Si, on which the LNO (200 nm) was fabricated. The quality of the NBT–BT film was characterized through morphology characterization by using an atomic force microscope (AFM; SII SPI-3700) and a field-emission scanning electron microscope (FE-SEM; HITACHI S4000).

### Phase identification and crystal structure analysis

The NBT–BT film was evaluated by four-circle X-ray diffraction (XRD; PANalytical X’Pert PRO MRD) to identify crystalline phase and crystal structure. To investigate the crystal lattice variation with respect to *E*-field, *in-situ E*-field dependent XRD was carried out at BL16XU of SPring-8. The sample size was 10 mm^2^ square, and the X-ray beam was irradiated on the Au top electrode (100 nm) deposited on the surface of the film, applying voltage to the bottom electrode and the top electrode was connected to ground. Electron diffraction (ED) measurement was done by utilizing a transmission electron microscope (TEM; JEOL JEM-2010F).

### Electrical property measurements

Ferro- and piezoelectric properties were examined using a ferroelectric test system (Radiant Technologies Precision Workstation). For piezoelectric property measurements the film was assessed in the form of a unimorph cantilever beam with a metal–insulator–metal electrode structure of which the top electrode was Au. The dimension of the beam was 2 mm (width) × 18 mm (length) and 0.5 mm (thickness). To estimate the transverse piezoelectricity, the displacement (δ) of the free end of the cantilever was recorded with a laser displacement meter (MTI MTI 2000) as a function of the *E*-field applied to the bottom electrode. Dielectric permittivity and loss were measured with an impedance analyzer (Agilent 4294 A).

## Electronic supplementary material

Supplementary Information
