# Supplementary material for: High electromechanical strain and enhanced temperature characteristics in lead-free (Na,Bi)TiO3–BaTiO3 thin films on Si substrates
Source: Sci Rep. 2018 May 18;8:7847. doi: 10.1038/s41598-018-26309-4 (PMC5959895; doi:10.1038/s41598-018-26309-4)
Supplement: Supplementary file 1 — Supplementary Information [file 41598_2018_26309_MOESM1_ESM.docx]

*Scientific Reports*

**High electromechanical strain and enhanced temperature characteristics in lead-free (Na,Bi)TiO_3_−BaTiO_3_ thin films on Si substrates**

Yoshiaki Tanaka^1^, Shoji Okamoto^1^, Kazuya Hashimoto^1^, Ryoichi Takayama^1^,

Takakiyo Harigai^1^, Hideaki Adachi^2^, and Eiji Fujii^2^

^1^*Engineering Division, Automotive & Industrial Systems Company, Panasonic Corporation,*

*1006 Kadoma, Kadoma City, Osaka 571-8501, Japan*

^2^*Advanced Research Division, Panasonic Corporation, 1006 Kadoma, Kadoma City, Osaka 571-8501, Japan*

**Supplementary information**

The microscopic crystalline structures in the polycrystalline thin film of (1-*x*)NBT–*x*BT with *x* = 0.07 on Si were investigated using AFM and SEM microscopy. As seen from Figs. 1S (a) and (b), the NBT–BT thin film had a smooth surface with root-mean-square (RMS) roughness of 3.3 nm over the 5.0 × 5.0 μm^2^ scanning area and a dense structure with columnar grains. Despite the high density of the film, no micro-cracks were identified, suggesting that the tensile thermal stress applied to the films is effectively counteracted.

**Figure S1 Microscopic crystalline structure** **for the NBT–BT thin film.** (a) AFM surface and (b) SEM cross sectional images.


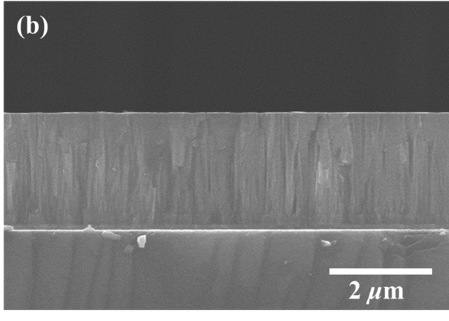

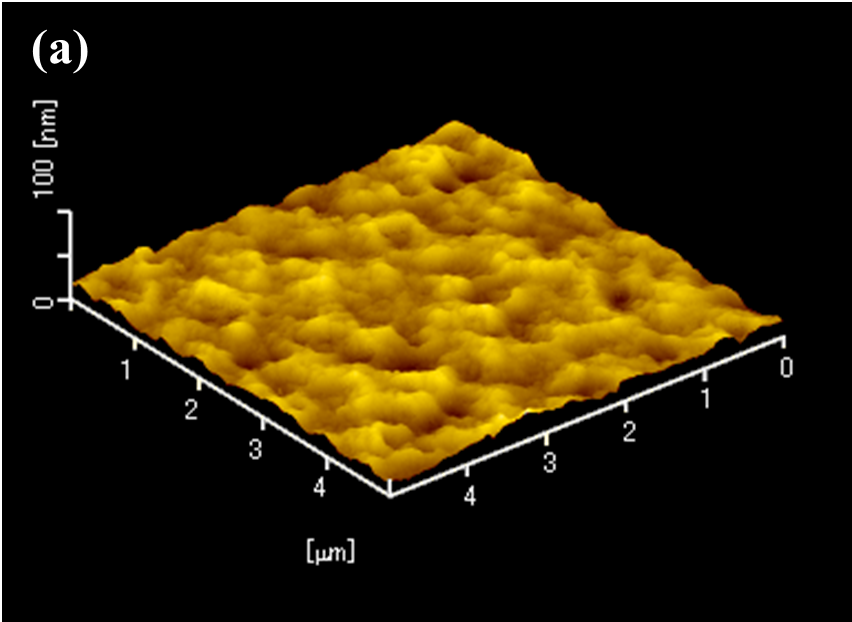


***Y. Tanaka et al; FIG. S1(a),(b)***
